# 5,8-Dibromo-2,11-dithia­[3,3](2,6)pyridino­paracyclo­phane

**DOI:** 10.1107/S1600536810028850

**Published:** 2010-07-24

**Authors:** Si-Si Li, Bei Zhang, Hong-Lin Zhang

**Affiliations:** a Key Laboratory of Pesticides and Chemical Biology of the Ministry of Education, College of Chemistry, Central China Normal University, Wuhan 430079, People’s Republic of China

## Abstract

The title compound, C_15_H_13_Br_2_NS_2_ [systematic name: 1^2^,1^5^-dibromo-2,7-dithia-1(1,4)-benzena-5(2,6)-pyridinaocta­phane], contains a dibromo-substituted benzene ring and a pyridine ring that are linked by a pair of bridging —CH_2_SCH_2_— groups. There is a weak π–π inter­action between the rings, the distance between the ring centroids being 3.572 (4) Å. The rings are not parallel, but form a dihedral angle of 18.29 (4)°.

## Related literature

For the preparation of the title compound, see: Kay & Baek (1997 ▶); Scheytza *et al.* (1999 ▶); Xu *et al.* (2008 ▶). For further information on paracyclo­phane and its derivatives, see: Wang *et al.* (2006 ▶); Yamamoto *et al.* (1997 ▶).
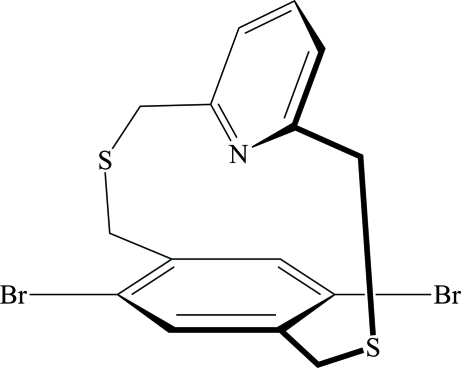


## Experimental

### 

#### Crystal data

C_15_H_13_Br_2_NS_2_
*M* *_r_* = 431.20
Monoclinic, 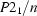

*a* = 8.9275 (15) Å
*b* = 18.879 (3) Å
*c* = 9.3213 (15) Å
β = 103.878 (3)°
*V* = 1525.2 (4) Å^3^
*Z* = 4
Mo *K*α radiation
μ = 5.58 mm^−1^
*T* = 298 K
0.16 × 0.12 × 0.10 mm

#### Data collection

Bruker SMART CCD area-detector diffractometer
Absorption correction: multi-scan (*SADABS*; Sheldrick, 1996 ▶) *T*_min_ = 0.452, *T*_max_ = 0.573
11428 measured reflections
3775 independent reflections
3018 reflections with *I* > 2σ(*I*)
*R*_int_ = 0.026

#### Refinement

*R*[*F* ^2^ > 2σ(*F* ^2^)] = 0.031
*wR*(*F* ^2^) = 0.078
*S* = 1.03
3775 reflections
181 parameters
H-atom parameters constrained
Δρ_max_ = 0.56 e Å^−3^
Δρ_min_ = −0.46 e Å^−3^

### 

Data collection: *SMART* (Bruker, 1997 ▶); cell refinement: *SAINT* (Bruker, 1999 ▶); data reduction: *SAINT*; program(s) used to solve structure: *SHELXS97* (Sheldrick, 2008 ▶); program(s) used to refine structure: *SHELXL97* (Sheldrick, 2008 ▶); molecular graphics: *SHELXTL* (Sheldrick, 2008 ▶); software used to prepare material for publication: *SHELXTL*.

## Supplementary Material

Crystal structure: contains datablocks I, global. DOI: 10.1107/S1600536810028850/pk2253sup1.cif

Structure factors: contains datablocks I. DOI: 10.1107/S1600536810028850/pk2253Isup2.hkl

Additional supplementary materials:  crystallographic information; 3D view; checkCIF report

## Figures and Tables

**Fig. 1.**
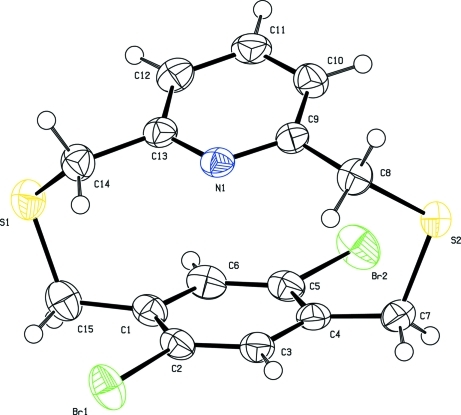
A view of the title compound, showing the atom-labelling scheme, with displacement ellipsoids drawn at the 50% probability level.

## supplementary crystallographic information

### Comment

In our research, we have synthesized the title compound 5,8-dibromo-2,11-dithia[3,3] (2,6)pyridinoparacyclophane. In the crystal structure, there are no classical hydrogen bonds, but is a weak π–π interaction. The distance between the centroid of the pyridine ring and the centroid of the benzene ring is 3.574 (4) Å, in addition, the angle between pyridine ring and the benzene ring is 18.29 (4)°.

### Experimental

The title compound was synthesized according to a modified literature procedure (Scheytza *et al.*, 1999).

A solution with equimolar amounts of the dithiol(3.26g, 10mmol) and 2,6-bis(bromomethyl) pyridine in degassed THF (500 mL) was added dropwise under N_2_ over 12 h to a refluxing solution of K_2_CO_3_(6.9g,50mmol) in EtOH (1.5 L).  After an additional 2h at the reflux temperature, the mixture was cooled and the solvent. The resulting residue was treated with CH_2_Cl_2_ (500 mL) and water (500mL). The organic phase was separated, and the aqueous layer extracted with CH_2_Cl_2_ three times. The combined organic layers were dried over MgSO_4_, then the solvent was removed, and the resulting solid was chromatographed on silica gel using CH_2_Cl_2_ as eluent. Colourless single crystals of the title compound suitable for X-ray diffraction were obtained by slow evaporation of a dichloromethane-n-hexane (1:30) solution over a period of 6 hours.

### Refinement

All H atoms were initially located in a difference map, but were constrained to an idealized geometry.  Constrained bond lengths and isotropic displacement parameters: (C—H =0.93 Å) and *U*_iso_(H) =1.2*U*_eq_(C) for aromatic H atoms, and (C—H =0.97 Å) and *U*_iso_(H) =1.2*U*_eq_(C) for methylene.

### Crystal data

**Table:**

C_15_H_13_Br_2_NS_2_	*F*(000) = 848
*M**_r_* = 431.20	*D*_x_ = 1.878 Mg m^−^^3^
Monoclinic, *P*2_1_/*n*	Mo *K*α radiation, λ = 0.71073 Å
Hall symbol: -P 2yn	Cell parameters from 4039 reflections
*a* = 8.9275 (15) Å	θ = 2.5–27.8°
*b* = 18.879 (3) Å	µ = 5.58 mm^−^^1^
*c* = 9.3213 (15) Å	*T* = 298 K
β = 103.878 (3)°	Block, colorless
*V* = 1525.2 (4) Å^3^	0.16 × 0.12 × 0.10 mm
*Z* = 4	

### Data collection

**Table:**

Bruker SMART CCD area-detector diffractometer	3775 independent reflections
Radiation source: fine-focus sealed tube	3018 reflections with *I* > 2σ(*I*)
graphite	*R*_int_ = 0.026
φ and ω scans	θ_max_ = 28.3°, θ_min_ = 2.2°
Absorption correction: multi-scan (*SADABS*; Sheldrick, 1996)	*h* = −11→11
*T*_min_ = 0.452, *T*_max_ = 0.573	*k* = −25→25
11428 measured reflections	*l* = −10→12

### Refinement

**Table:**

Refinement on *F*^2^	Primary atom site location: structure-invariant direct methods
Least-squares matrix: full	Secondary atom site location: difference Fourier map
*R*[*F*^2^ > 2σ(*F*^2^)] = 0.031	Hydrogen site location: inferred from neighbouring sites
*wR*(*F*^2^) = 0.078	H-atom parameters constrained
*S* = 1.03	*w* = 1/[σ^2^(*F*_o_^2^) + (0.0414*P*)^2^ + 0.2049*P*] where *P* = (*F*_o_^2^ + 2*F*_c_^2^)/3
3775 reflections	(Δ/σ)_max_ = 0.002
181 parameters	Δρ_max_ = 0.56 e Å^−^^3^
0 restraints	Δρ_min_ = −0.46 e Å^−^^3^

### Special details

**Table:**

Geometry. All e.s.d.'s (except the e.s.d. in the dihedral angle between two l.s. planes) are estimated using the full covariance matrix. The cell e.s.d.'s are taken into account individually in the estimation of e.s.d.'s in distances, angles and torsion angles; correlations between e.s.d.'s in cell parameters are only used when they are defined by crystal symmetry. An approximate (isotropic) treatment of cell e.s.d.'s is used for estimating e.s.d.'s involving l.s. planes.
Refinement. Refinement of *F*^2^ against ALL reflections. The weighted *R*-factor *wR* and goodness of fit *S* are based on *F*^2^, conventional *R*-factors *R* are based on *F*, with *F* set to zero for negative *F*^2^. The threshold expression of *F*^2^ > σ(*F*^2^) is used only for calculating *R*-factors(gt) *etc*. and is not relevant to the choice of reflections for refinement. *R*-factors based on *F*^2^ are statistically about twice as large as those based on *F*, and *R*- factors based on ALL data will be even larger.

### Fractional atomic coordinates and isotropic or equivalent isotropic displacement parameters (Å^2^)

**Table:**

	*x*	*y*	*z*	*U*_iso_*/*U*_eq_	
Br1	0.63099 (3)	0.048688 (14)	0.87832 (3)	0.04897 (10)	
Br2	0.22924 (4)	0.139645 (16)	0.22281 (3)	0.05124 (10)	
C1	0.3799 (3)	0.03373 (12)	0.6206 (3)	0.0338 (5)	
C2	0.5079 (3)	0.07344 (12)	0.6886 (3)	0.0334 (5)	
C3	0.5520 (3)	0.13348 (12)	0.6246 (3)	0.0331 (5)	
H3	0.6384	0.1587	0.6742	0.040*	
C4	0.4686 (3)	0.15680 (13)	0.4867 (3)	0.0324 (5)	
C5	0.3444 (3)	0.11522 (13)	0.4148 (3)	0.0338 (5)	
C6	0.3002 (3)	0.05574 (12)	0.4802 (3)	0.0372 (5)	
H6	0.2154	0.0298	0.4295	0.045*	
C7	0.5137 (3)	0.22521 (13)	0.4279 (3)	0.0383 (6)	
H7A	0.5180	0.2181	0.3259	0.046*	
H7B	0.6168	0.2376	0.4835	0.046*	
C8	0.3410 (3)	0.28873 (14)	0.6143 (3)	0.0371 (5)	
H8A	0.4332	0.2727	0.6849	0.045*	
H8B	0.3134	0.3346	0.6471	0.045*	
C9	0.2123 (3)	0.23745 (12)	0.6156 (2)	0.0301 (5)	
C10	0.0700 (3)	0.24389 (14)	0.5179 (3)	0.0382 (5)	
H10	0.0501	0.2810	0.4505	0.046*	
C11	−0.0417 (3)	0.19455 (15)	0.5220 (3)	0.0414 (6)	
H11	−0.1389	0.1982	0.4580	0.050*	
C12	−0.0085 (3)	0.13961 (13)	0.6216 (3)	0.0387 (6)	
H12	−0.0819	0.1050	0.6243	0.046*	
C13	0.1362 (3)	0.13660 (11)	0.7180 (3)	0.0308 (5)	
C14	0.1781 (3)	0.07834 (13)	0.8309 (3)	0.0384 (6)	
H14A	0.1171	0.0840	0.9034	0.046*	
H14B	0.2858	0.0835	0.8820	0.046*	
C15	0.3230 (3)	−0.02833 (13)	0.6927 (3)	0.0426 (6)	
H15A	0.4036	−0.0430	0.7770	0.051*	
H15B	0.3036	−0.0674	0.6232	0.051*	
N1	0.2443 (2)	0.18540 (10)	0.7172 (2)	0.0302 (4)	
S1	0.14839 (8)	−0.01069 (3)	0.75438 (8)	0.04531 (17)	
S2	0.38563 (7)	0.29931 (3)	0.43559 (7)	0.03722 (15)	

### Atomic displacement parameters (Å^2^)

**Table:**

	*U*^11^	*U*^22^	*U*^33^	*U*^12^	*U*^13^	*U*^23^
Br1	0.05493 (19)	0.04386 (16)	0.03946 (16)	0.00297 (12)	−0.00566 (13)	0.01018 (11)
Br2	0.0613 (2)	0.05608 (19)	0.02904 (15)	0.00966 (13)	−0.00356 (12)	−0.00142 (11)
C1	0.0373 (13)	0.0296 (11)	0.0345 (13)	0.0066 (9)	0.0086 (10)	−0.0027 (10)
C2	0.0351 (13)	0.0354 (12)	0.0282 (12)	0.0097 (10)	0.0047 (10)	0.0013 (9)
C3	0.0287 (12)	0.0383 (12)	0.0322 (12)	0.0040 (9)	0.0072 (10)	0.0009 (10)
C4	0.0294 (12)	0.0381 (12)	0.0320 (12)	0.0091 (9)	0.0122 (10)	0.0014 (10)
C5	0.0348 (13)	0.0402 (12)	0.0257 (11)	0.0097 (10)	0.0059 (9)	−0.0032 (10)
C6	0.0368 (13)	0.0375 (12)	0.0351 (13)	0.0034 (10)	0.0045 (11)	−0.0088 (10)
C7	0.0337 (13)	0.0467 (14)	0.0379 (13)	0.0047 (10)	0.0150 (10)	0.0061 (11)
C8	0.0401 (14)	0.0384 (13)	0.0332 (13)	−0.0020 (10)	0.0095 (11)	−0.0027 (10)
C9	0.0348 (12)	0.0311 (11)	0.0268 (11)	0.0025 (9)	0.0123 (9)	−0.0041 (9)
C10	0.0388 (13)	0.0422 (13)	0.0330 (13)	0.0072 (11)	0.0075 (10)	0.0056 (10)
C11	0.0263 (12)	0.0571 (16)	0.0394 (14)	0.0082 (11)	0.0049 (10)	0.0043 (12)
C12	0.0312 (13)	0.0447 (14)	0.0404 (14)	−0.0040 (10)	0.0092 (11)	−0.0022 (11)
C13	0.0314 (12)	0.0345 (12)	0.0283 (12)	0.0030 (9)	0.0105 (9)	−0.0032 (9)
C14	0.0462 (15)	0.0374 (13)	0.0332 (13)	0.0031 (11)	0.0122 (11)	0.0017 (10)
C15	0.0517 (16)	0.0303 (12)	0.0441 (15)	0.0008 (11)	0.0082 (12)	−0.0017 (11)
N1	0.0309 (10)	0.0332 (10)	0.0277 (10)	0.0038 (7)	0.0093 (8)	−0.0038 (8)
S1	0.0464 (4)	0.0343 (3)	0.0559 (4)	−0.0053 (3)	0.0138 (3)	0.0052 (3)
S2	0.0394 (3)	0.0376 (3)	0.0363 (3)	0.0021 (2)	0.0123 (3)	0.0090 (3)

### Geometric parameters (Å, °)

**Table:**

Br1—C2	1.903 (2)	C8—H8B	0.9700
Br2—C5	1.895 (2)	C9—N1	1.347 (3)
C1—C2	1.386 (3)	C9—C10	1.379 (3)
C1—C6	1.395 (3)	C10—C11	1.371 (4)
C1—C15	1.498 (4)	C10—H10	0.9300
C2—C3	1.381 (3)	C11—C12	1.377 (4)
C3—C4	1.393 (3)	C11—H11	0.9300
C3—H3	0.9300	C12—C13	1.386 (3)
C4—C5	1.392 (3)	C12—H12	0.9300
C4—C7	1.496 (3)	C13—N1	1.335 (3)
C5—C6	1.380 (3)	C13—C14	1.506 (3)
C6—H6	0.9300	C14—S1	1.820 (3)
C7—S2	1.819 (3)	C14—H14A	0.9700
C7—H7A	0.9700	C14—H14B	0.9700
C7—H7B	0.9700	C15—S1	1.817 (3)
C8—C9	1.505 (3)	C15—H15A	0.9700
C8—S2	1.814 (2)	C15—H15B	0.9700
C8—H8A	0.9700		
			
C2—C1—C6	116.5 (2)	N1—C9—C10	122.3 (2)
C2—C1—C15	123.3 (2)	N1—C9—C8	116.2 (2)
C6—C1—C15	120.2 (2)	C10—C9—C8	121.5 (2)
C3—C2—C1	122.4 (2)	C11—C10—C9	118.9 (2)
C3—C2—Br1	116.22 (18)	C11—C10—H10	120.6
C1—C2—Br1	121.40 (18)	C9—C10—H10	120.6
C2—C3—C4	120.9 (2)	C10—C11—C12	119.3 (2)
C2—C3—H3	119.6	C10—C11—H11	120.3
C4—C3—H3	119.6	C12—C11—H11	120.3
C5—C4—C3	117.0 (2)	C11—C12—C13	119.0 (2)
C5—C4—C7	124.3 (2)	C11—C12—H12	120.5
C3—C4—C7	118.6 (2)	C13—C12—H12	120.5
C6—C5—C4	121.7 (2)	N1—C13—C12	122.0 (2)
C6—C5—Br2	117.85 (18)	N1—C13—C14	116.7 (2)
C4—C5—Br2	120.44 (19)	C12—C13—C14	121.3 (2)
C5—C6—C1	121.4 (2)	C13—C14—S1	114.34 (18)
C5—C6—H6	119.3	C13—C14—H14A	108.7
C1—C6—H6	119.3	S1—C14—H14A	108.7
C4—C7—S2	115.00 (17)	C13—C14—H14B	108.7
C4—C7—H7A	108.5	S1—C14—H14B	108.7
S2—C7—H7A	108.5	H14A—C14—H14B	107.6
C4—C7—H7B	108.5	C1—C15—S1	114.02 (17)
S2—C7—H7B	108.5	C1—C15—H15A	108.7
H7A—C7—H7B	107.5	S1—C15—H15A	108.7
C9—C8—S2	114.50 (16)	C1—C15—H15B	108.7
C9—C8—H8A	108.6	S1—C15—H15B	108.7
S2—C8—H8A	108.6	H15A—C15—H15B	107.6
C9—C8—H8B	108.6	C13—N1—C9	118.4 (2)
S2—C8—H8B	108.6	C15—S1—C14	103.75 (12)
H8A—C8—H8B	107.6	C8—S2—C7	103.29 (12)
			
C6—C1—C2—C3	2.6 (3)	S2—C8—C9—C10	−53.5 (3)
C15—C1—C2—C3	−175.3 (2)	N1—C9—C10—C11	−1.6 (4)
C6—C1—C2—Br1	−178.55 (17)	C8—C9—C10—C11	178.4 (2)
C15—C1—C2—Br1	3.6 (3)	C9—C10—C11—C12	−0.8 (4)
C1—C2—C3—C4	−0.2 (4)	C10—C11—C12—C13	1.5 (4)
Br1—C2—C3—C4	−179.17 (17)	C11—C12—C13—N1	0.1 (4)
C2—C3—C4—C5	−2.8 (3)	C11—C12—C13—C14	179.1 (2)
C2—C3—C4—C7	175.2 (2)	N1—C13—C14—S1	−126.8 (2)
C3—C4—C5—C6	3.5 (3)	C12—C13—C14—S1	54.2 (3)
C7—C4—C5—C6	−174.3 (2)	C2—C1—C15—S1	105.8 (2)
C3—C4—C5—Br2	−177.02 (16)	C6—C1—C15—S1	−71.9 (3)
C7—C4—C5—Br2	5.1 (3)	C12—C13—N1—C9	−2.4 (3)
C4—C5—C6—C1	−1.2 (4)	C14—C13—N1—C9	178.6 (2)
Br2—C5—C6—C1	179.31 (18)	C10—C9—N1—C13	3.2 (3)
C2—C1—C6—C5	−1.8 (3)	C8—C9—N1—C13	−176.80 (19)
C15—C1—C6—C5	176.1 (2)	C1—C15—S1—C14	−40.3 (2)
C5—C4—C7—S2	72.8 (3)	C13—C14—S1—C15	83.7 (2)
C3—C4—C7—S2	−105.0 (2)	C9—C8—S2—C7	−83.4 (2)
S2—C8—C9—N1	126.56 (19)	C4—C7—S2—C8	40.9 (2)
